# Schockraum- und Schwerverletztenversorgung als „Verlustgeschäft“: Erfahrungen eines Schweizer Traumazentrums im aktuellen DRG-System

**DOI:** 10.1007/s00113-020-00937-w

**Published:** 2020-12-18

**Authors:** Thomas Gross, Felix Amsler

**Affiliations:** 1 Klinik für Traumatologie, Kantonsspital Aarau, Tellstr. 1, 5001 Aarau, Schweiz; 2 Klinik für Orthopädie und Traumatologie des Bewegungsapparates, Kantonsspital Baselland, 4101 Bruderholz, Schweiz; 3 Amsler Consulting, Gundeldingerrain 111, 4059 Basel, Schweiz

**Keywords:** Krankenhauskosten, Diagnosis Related Groups (DRG), Notfall, Trauma, Gesundheitsökonomie, Economics, Hospital costs, Diagnosis Related Groups (DRG), Trauma center, Health insurance reimbursement

## Abstract

**Hintergrund:**

Es galt herauszufinden, wie kostendeckend die Versorgung potenziell Schwerverletzter in einem Schweizer Traumazentrum ist, und inwieweit Spitalgewinne bzw. -verluste mit patientenbezogenen Unfall‑, Behandlungs- oder Outcome-Daten korrelieren.

**Methodik:**

Analyse aller 2018 im Schockraum (SR) bzw. mit Verletzungsschwere New Injury Severity Score (NISS) ≥8 notfallmäßig stationär behandelter Patienten eines Schwerverletztenzentrums der Schweiz (uni- und multivariate Analyse; *p* < 0,05).

**Ergebnisse:**

Für das Studienkollektiv (*n* = 513; Ø NISS = 18) resultierte gemäß Spitalkostenträgerrechnung ein Defizit von 1,8 Mio. CHF. Bei einem Gesamtdeckungsgrad von 86 % waren 66 % aller Fälle defizitär (71 % der Allgemein- vs. 42 % der Zusatzversicherten; *p* < 0,001). Im Mittel betrug das Defizit 3493.- pro Patient (allg. Versicherte, Verlust 4545.-, Zusatzversicherte, Gewinn 1318.-; *p* < 0,001). Auch „in“- und „underlier“ waren in 63 % defizitär. SR-Fälle machten häufiger Verlust als Nicht-SR-Fälle (73 vs. 58 %; *p* = 0,002) wie auch Traumatologie- vs. Neurochirurgiefälle (72 vs. 55 %; *p* < 0,001). In der multivariaten Analyse ließen sich 43 % der Varianz erhaltener Erlöse mit den untersuchten Variablen erklären. Hingegen war der ermittelte Deckungsgrad nur zu 11 % (korr. R^2^) durch die Variablen SR, chirurgisches Fachgebiet, Intensivaufenthalt, Thoraxverletzungsstärke und Spitalletalität zu beschreiben. Case-Mix-Index gemäß aktuellen Diagnosis Related Groups (DRG) und Versicherungsklasse addierten weitere 13 % zu insgesamt 24 % erklärter Varianz.

**Diskussion:**

Die notfallmäßige Versorgung potenziell Schwerverletzter an einem Schweizer Traumazentrum erweist sich nur in einem Drittel der Fälle als zumindest kostendeckend, dies v. a. bei Zusatzversicherten, Patienten mit einem hohen Case-Mix-Index oder einer IPS- bzw. kombinierten Polytrauma- und Schädel-Hirn-Trauma-DRG-Abrechnungsmöglichkeit.

**Zusatzmaterial online:**

Die Online-Version dieses Beitrags (10.1007/s00113-020-00937-w) enthält weitere Tabellen und Abbildungen (s. Verweise „Zusatzmaterial online: Abb.“ bzw. „Zusatzmaterial online: Tab.“ im Text). Beitrag und Zusatzmaterial stehen Ihnen auf www.springermedizin.de zur Verfügung. Bitte geben Sie dort den Beitragstitel in die Suche ein, das Zusatzmaterial finden Sie beim Beitrag unter „Ergänzende Inhalte“.

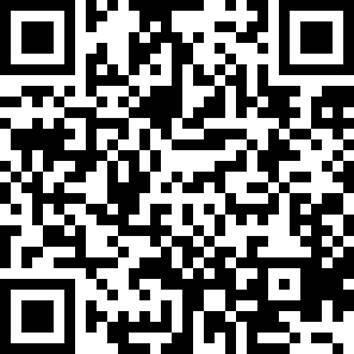

## Abstract (en)

**Background:**

The study objective was to find out how cost-covering the treatment of patients with a potentially severe injury actually is in a Swiss trauma center and to what extent hospital profits/losses correlate with patient-related accident, treatment and outcome variables.

**Methods:**

Analysis of all patients hospitalized in a Swiss trauma center in 2018 following treatment in the emergency room (ER) and/or with a significant injury (new injury severity score, NISS ≥8). Hospital cost-benefit calculation using current Swiss diagnosis-related groups (DRG) and the REKOLE© billing system (univariate and multivariate analysis; *p* < 0.05).

**Results:**

From a hospital point of view, the study cohort (*n* = 513; Ø NISS = 18) generated a deficit of 1.8 million CHF. This corresponded to a total coverage of 86%, with 66% of cases incurring a loss (71% of statutory insurance vs. 42% of privately insured; *p* < 0.001). On average, the deficit was 3493 CHF per patient (4545 CHF for statutory insurance vs. 1318 CHF for privately insured; *p* < 0.001), with a loss also in 63% of inliers and underliers (DRG). The ER cases more frequently caused a financial loss than non-ER cases (73% vs. 58%; *p* = 0.002) or traumatology vs. neurosurgery cases (72% vs. 55%; *p* < 0.001). In multivariable analysis 43% of the variance of financial returns were explained by the studied parameters. In contrast, only 11% (adjusted R^2^) of the variance of the hospital cover ratio could be described by the variables ER, surgical specialty, intensive care, thoracic injury and hospital mortality. The case mix index (DRG) and type of insurance added a further 13% to a total of 24% explained variance.

**Conclusion:**

From a Swiss trauma center point of view only one third of emergencies are not loss-making, most of all privately insured patients or cases billable via a combined polytrauma and head trauma DRG.

## Hinführung zum Thema

International wird die Schwerverletztenversorgung meist als nichtkostentragend angesehen [12, 21, 26, 27]. Die „Diagnosis-related-groups“(DRG)-Entlohnung erbrachte diesbezüglich Verbesserungen [7, 9, 17, 29, 33], und Verluste wurden v. a. noch bei Schwerverletzten (z. B. mit Injury Severity Score [ISS] >15) beschrieben [12]. Dies z. B. für Deutschland trotz in den letzten Jahren erfolgter DRG-Anpassungen [12], andererseits wurden aber auch neu kostendeckende Verhältnisse, teilweise sogar Überfinanzierungen, nachgewiesen [24]. Allerdings fehlen aktuell Daten für den gesamten Versorgungsauftrag eines Traumazentrums, d. h. auch unter Einbezug des Aufwands routinemäßiger Schockraum(SR)-Versorgung weniger schwer Verletzter [25]. Aufgrund eigener Beobachtungen sowie neuerer Literaturhinweise, dass v. a. die SR-Behandlung defizitär sein könnte [25, 34], unternahmen wir diese Studie.

## Hintergrund und Fragestellung

Bereits in den 1980er-Jahren realisierten US-amerikanische Traumazentren zunehmende finanzielle Verluste infolge einer steigenden Zahl zu versorgender Schwerverletzter [31]. Erste publizierte Kostenanalysen zeigten auch in Deutschland für die Behandlung des Polytraumas hohe wirtschaftliche Verluste der erstversorgenden Kliniken, weshalb Sonderentgelte gefordert wurden [28]. Frühere Untersuchungen beschränkten sich mehrheitlich auf das Polytrauma bzw. Schwerverletzte (z. B. ISS >15) [6, 17, 27–30, 32], die SR-Versorgung [13], spezifische Verletzungsmuster wie Verbrennung [26] und Schädel-Hirn-Trauma (SHT) [34] oder das Trauma allgemein [9, 23, 31].

Gewinne bzw. Verluste und damit der Kostendeckungsgrad einer Behandlung resultieren aus entsprechenden Kosten wie Erträgen pro Fall. Allerdings erfolgt v. a. im stationären Bereich eine Erfassung des je nach Spital und Gesundheitssystem stark variierenden Aufwands selbst in hochindustrialisierten Ländern routinemäßig allein mittels Kostendatenerhebung, wobei länder- und regionenspezifisch verschiedene Kostenträgersysteme seitens jeweiligem Krankenhaus-Controlling zur Anwendung kommen. Bzgl. der Ertragsseite benutzen inzwischen viele Länder ein pauschaliertes Entgeltsystem Typ DRG zur Abrechnung stationärer Patienten [24, 29]. Historisch gesehen importierten z. B. Deutschland und Frankreich ihre DRG-Systeme zwischen 1995 und 2005 ursprünglich aus den USA [23] und Australien [4, 8], die Schweiz 2012 sodann aus Deutschland [10]. Ungeachtet ihrer gemeinsamen Wurzeln wurden die jeweiligen DRG-Entgelte allerdings länderspezifischen Gegebenheiten angepasst, was die internationale Vergleichbarkeit wiederum erschwert.

Trotz verschiedener Arbeiten zur sachgerechten Vergütung spezifischer Verletzungsbehandlungen gerade auch im deutschsprachigen Raum konnten wir in der Literatur keine detaillierte Arbeit bzgl. der „Rentabilität“ des Versorgungsauftrages eines Traumazentrums nicht nur bzgl. alleiniger Schwerverletztenversorgung, sondern auch unter Einschluss routinemäßiger SR-Behandlung bzw. weniger schwer Verletzter finden.

Vor dem Hintergrund dieser fehlenden bzw. widersprüchlichen Angaben zur aktuellen Kostenertragslage typischer Traumazentrumversorgung führten wir diese prospektive Studie mit folgenden Hauptfragestellungen durch:

1. Wie kostendeckend ist die notfallmäßige Versorgung bei Verdacht auf Schwerverletzung, d. h. im SR Behandelter bzw. signifikant Verletzter, eines mitteleuropäischen Zentrumspitals bzgl. aktueller (Swiss‑)DRG-Entgelte?
2. Wie unterscheiden sich Kosten, Erlöse und Deckungsbeiträge je nach alltagsrelevanten Behandlungsgruppen?
3. Inwieweit erklären Patienten‑, Unfall‑, Behandlungs‑, Kurzzeit-Outcome-Daten sowie der Versicherungsstatus resultierende Gewinne bzw. Verluste?

## Methodik

### Studiendesign und Datenerhebung

Konsekutive Analyse aller 2018 im SR gemäß standardisierten Kriterien [3] und/oder infolge eines signifikanten Traumas (New Injury Severity Score, NISS ≥8) [15] notfallmäßig (innert 24 h) stationär versorgter Verletzten eines Schweizer Zentrumspitals. Die Studie war Teil eines von der zuständigen Ethikkommission (PB_2018-00079 – AG/SO 2012-008) genehmigten Versorgungsforschungsprojektes (NCT02165137). Die prospektive klinische Datenregistrierung umfasste detaillierte Angaben zu Demografie, Unfallmechanismus, Spitalprozessen und Outcome, analog der Eingabe im TraumaRegisterDGU® (http://www.traumaregister-dgu.de). Die Graduierung der Traumaschwere wurde gemäß Abbreviated Injury Scale (AIS) bzw. Injury Severity Score (ISS) und New ISS (NISS), in der modifizierten Version 2005 der DGU, von speziell geschulten „study nurses“ vorgenommen. Schwerverletzte wurden gemäß Kriterien der hochspezialisierten Medizin (HSM) der Schweiz definiert: ISS >19 (bzw. >15 bei Kindern) und/oder AIS Schädel-Hirn >2 [15] (Zusatzmaterial online: Tab. A). Die Kostendatenerhebung erfolgte gemäß administrativer Erfassung des Krankenhaus-Controlling, unter Benutzung aktueller DRG-Codierung (https://www.swissdrg.org/de/akutsomatik/swissdrg) und der schweizweiten Spitalkostenträgerrechnung REKOLE© (Revision der Kosten- und Leistungserfassung, Schweizer Standard zur Erhebung der entstandenen Kosten im Krankenhaus; https://www.hplus.ch/de/rechnungswesen/handbuch-rekole/): Auf der Einnahmenseite stehen „Erlöse“: Gesamtspitalerlöse pro Fall (inkl. Zusatzversicherungsanteil) und „DRG-Erlöse“: Erlöse gemäß Swiss-DRG 2018. Auf der Kostenseite stehen „Einzelkosten“, „Gemeinkosten“ und „Anlagenutzungskosten“ (Kostenmatrix: Zusatzmaterial online: Tab. B), welche zusammen die Gesamtkosten nach REKOLE© ausmachen. Daraus werden Verlust bzw. Gewinn (DB III = Erlöse minus Gesamtkosten) berechnet (Zusatzmaterial online: Tab. C). Zusätzlich wurden der Gesamtdeckungsgrad (Gesamterlöse/Gesamtkosten) und der Anteil der Patienten mit Verlust berechnet. Eingeschlossen wurden Patienten, welche im selben Jahr ein- und austraten. Die jeweiligen Haupt-DRG pro Fall wurden in 6 Untergruppen kategorisiert: Polytrauma (PT: W02, W60, W61); Intensivmedizinische Komplexbehandlung oder PT & SHT: (W01, W36, A07, A11, A13, A36, B36, E36, E40, E64, F36, F43, T36, T60); SHT: (B09, B78, B79, B80); Wirbelsäulenverletzung (WS: B02, B20, D28, B61, I06, I09, I19, I68); Extremitätenverletzung (Extr: I08, I13, I16, I18, I20, I21, I30, I31, I32, I57, I59, I05, I29, I74, I75, I77, I78) sowie andere Verletzungen (übrige DRG).

### Statistische Auswertung

Statistische Tests wurden zweiseitig durchgeführt mittels Student’s *t*-Tests für Mittelwertsvergleiche zwischen 2 Gruppen. Für die Signifikanzberechnung des Deckungsgrades wurden die logarithmierten und dadurch normal verteilten Werte des Deckungsgrades pro Patient verwendet. Beim Vergleich derselben Kostenvariablen zwischen verschiedenen Gruppen wurden die Signifikanzen Bonferroni-korrigiert, d. h., bei mehr als 2 Gruppen wurden Varianzanalysen (ANOVA) mit Post-hoc-Tests mit Bonferroni-Korrektur gerechnet. Für kategoriale Daten wurden Chi-Quadrat-Tests verwendet. Um den univariaten Einfluss der Patientenvariablen auf die Kostendaten zu überprüfen, wurde eine nach Zusatzversicherung partialisierte Pearson-Korrelationsanalyse verwendet. Multivariat wurden block- und schrittweise lineare Regressionen gerechnet (Einschlusskriterium *p* < 0,05; Ausschluss *p* > 0,1). Ins Modell aufgenommen wurden in einem ersten Block relevante Patienten‑, Verletzungs- und Behandlungsvariablen (Alter, Sekundärverlegung; SR-Fall, AIS Schädel, AIS Regionen 2–6, AIS Extremitäten, hauptverantwortliche bzw. fallführende Fachdisziplin Traumatologie bzw. Neurochirurgie, IPS-Aufenthalt, Durchführungen von Operationen) in einem zweiten Block die Outcome-Variablen stationärer Rehabilitationsaufenthalt nach Spitalentlassung und Verstorben im Spital sowie in einem dritten Block der Case-Mix-Index und das Vorhandensein einer Zusatzversicherung. Die statistischen Analysen erfolgten mit SPSS^TM^ für Windows 26 (Armonk, NY: IBM Corp, USA), *p*-Werte <0,05 wurden als signifikant definiert.

## Ergebnisse

### Studienkollektiv und Versicherungsstatus

Im Gesamtstudienkollektiv (*n* = 513; ISS 13,3; Zusatzmaterial online: Tab. A) standen Gesamterlöse von CHF 21.480.- pro Patient Gesamtausgaben von 24.970.- gegenüber (Tab. 1). Der resultierende durchschnittliche Verlust von 3490.- entsprach einem Deckungsgrad von 86 %. 82 % der Verletzten waren allgemein- und 18 % zusatzversichert und wiesen eine vergleichbare erwartete Letalität (RISC2) auf (Zusatzmaterial online: Tab C). Die Gesamterlöse waren bei Zusatzversicherten fast doppelt so hoch wie bei Allg.-Versicherten (34.340.- vs. 18.660.-; *p* < 0,001), was nach Abzug der jeweiligen Gesamtkosten zu einem Gewinn von 1320.- bei Zusatzversicherten (Deckungsgrad 104 %) bzw. Verlust von CHF 4550 bei Allg.-Versicherten (Deckungsgrad 80 %) führte. Damit machte das Spital bei 71 % der Allg.- bzw. 42 % der Zusatzversicherten einen finanziellen Verlust (*p* < 0,001).

**Table 1:** 

	Case-Mix-Index	DRG-Erlöse	*p*	Gesamterlöse	*p*	Gesamtkosten	*p*	DB III	*p*	Deckungsgrad	*p*	Patienten mit Verlust	*p*
Total (*n* = 513)	1,93 (2,26)	18.953 (22.109)	–	21.474 (24.676)	–	−24.968 (26.147)	–	−3493 (11.211)	–	86 %	–	66 %	–
Nicht schwer verletzt (*n* = 263)	1,69 (1,98)	16.591 (19.372)	0,078	18.639 (20.866)	**0,045**	−22.925 (24.203)	0,417	−4287 (9961)	0,602	81 %	**0,018**	70 %	0,174
Schwer verletzt, HSM (*n* = 250)	2,19 (2,5)	21.438 (24.457)		24.457 (27.867)		−27.117 (27.935)		−2659 (12.356)		90 %		61 %	
Primär versorgt (*n* = 357)	1,93 (2,39)	18.879 (23.456)	1,000	21.524 (25.628)	1,000	−25.464 (26.602)	1,000	−3940 (11.676)	1,000	85 %	**0,000**	71 %	**0,004**
Sekundär zuverlegt (*n* = 156)	1,95 (1,91)	19.124 (18.734)		21.360 (22.425)		−23.832 (25.122)		−2472 (10.028)		90 %		55 %	
Kein SR (*n* = 232)	1,69 (1,37)	16.552 (13.408)	0,152	19.213 (17.775)	0,356	−22.039 (20.933)	0,126	−2826 (8561)	1,000	87 %	**0,000**	58 %	**0,002**
SR (*n* = 281)	2,14 (2,77)	20.936 (27.144)		23.341 (29.070)		−27.386 (29.591)		−4045 (12.988)		85 %		73 %	
Traumatologie (*n* = 218)	1,83 (2,48)	17.939 (24.298)	0,284	20.716 (27.203)	0,342	−25.027 (29.918)	2,354	−4310 (11.224)	0,169	83 %	**0,000**	72 %	**0,000**
Neurochirurgie (*n* = 196)	2,19 (2,2)	21.414 (21.581)		24.111 (24.312)		−26.225 (23.817)		−2114 (11.838)		92 %		55 %	
Allgemein (*n* = 421)	1,86 (2,22)	18.193 (21.775)	0,573	18.663 (21.941)	**0,000**	−23.208 (24.737)	**0,006**	−4545 (10.499)	**0,000**	80 %	**0,000**	71 %	**0,000**
Zusatzversichert (*n* = 92)	2,29 (2,39)	22.434 (23.386)		34.339 (31.627)		−33.021 (30.699)		1318 (13.035)		104 %		42 %	
Under- oder Inlier (*n* = 432)	2,02 (2,34)	19.812 (22.956)	0,252	22.281 (24.858)	0,523	−25.144 (25.644)	1,000	−2862 (11.149)	**0,019**	89 %	**0,000**	63 %	**0,018**
Overlier (*n* = 81) gemäß DRG	1,47 (1,66)	14.373 (16.254)		17.171 (23.361)		−24.030 (28.835)		−6859 (11.004)		72 %		80 %	

Mittelwert (Standardabweichung) bzw. Prozent und je Bonferroni-korrigierte Signifikanz (*p*)

Signifikante Wertangaben (p < 0,05) sind fett dargestellt

*DB III* Deckungsbetrag („Verlust/Gewinn“) gemäß REKOLE©-Abrechnung, *SR* Schockraum, *DRG* Diagnosis Related Groups

### Kosten‑/Erlösdaten unter speziellen Konstellationen

**Figure 1:**
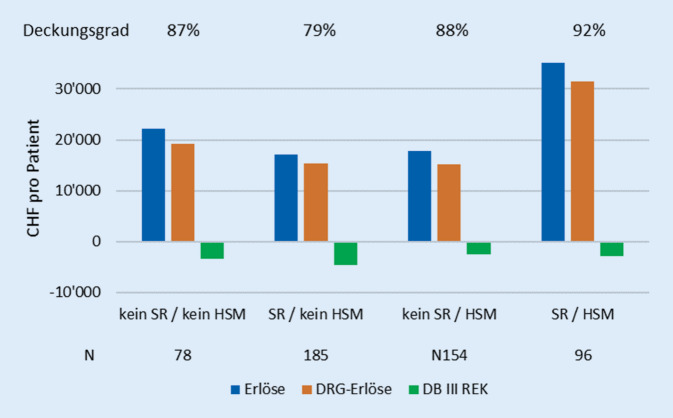

Die Behandlung Schwerverletzter (Zusatzmaterial online: Abb. A) generierte einen höheren Deckungsgrad von 90 % vs. 81 % im Vergleich mit Nichtschwerverletzten (*p* = 0,018). Direkt vom Unfallort eingewiesene Patienten zeigten einen niedrigeren Deckungsgrad als sekundär Zuverlegte (*p* < 0,001). Eine initiale SR-Versorgung erwies sich häufiger als defizitär als diejenige von Nicht-SR-Fällen (*p* < 0,001). Die kombinierte Analyse bzgl. SR-Versorgung und Schwerverletzung ließ einen additiven finanziellen Effekt dieser Konstellationen erkennen, mit dem tiefsten Deckungsgrad (79 %) für im SR versorgte Nichtschwerverletzte (Abb. 1 sowie Zusatzmaterial online: Tab. D). Einen signifikant geringeren Deckungsgrad (83 %) erreichte die Unfallchirurgie als hauptverantwortlich fallführende Fachdisziplin im Vergleich zur Neurochirurgie (92 %, *p* < 0,001; Tab. 1). Entsprachen bei Allg.-Versicherten die DRG-Erlöse nahezu vollständig den Gesamterlösen, so erbrachte eine Zusatzversicherung dem Krankenhaus im Mittel pro Patient ein Drittel mehr Erlöse (*p* < 0,001) und eine Erhöhung des Deckungsgrades von 80 auf 104 % (Tab. 1). In- und Underlier gemeinsam generierten eher höhere DRG-Erlöse als Overlier (n. s.), waren aber trotzdem durchschnittlich defizitär, wenn auch weniger (Deckungsgrad 89 % vs. 72 %, *p* < 0,001; Tab. 1). Der mittlere Case-Mix-Index betrug 1,93 (±2,26), was einem gesamten Case-Mix von 990 Punkten entsprach, wobei ein Case-Mix-Punkt im Untersuchungszeitraum CHF 9800,- ausmachte (regelhaft CHF 9700 für obligatorisch Krankenversicherte, CHF 10.000,- für Unfall‑, Militär- und Invalidenversicherte sowie CHF 12.000 für ausländische, Nicht-EU-Patienten).

**Figure 2:**
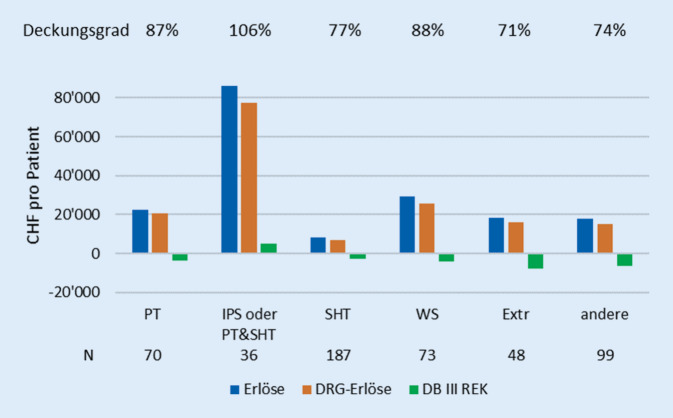

Die Kategorisierung nach Haupt-DRG zeigt die weitaus höchsten Erlöse für die schwerstverletzte Gruppe mit intensivmedizinischer Komplexbehandlung und/oder PT & SHT (*n* = 36, 7 % aller Patienten). Dies ist auch die einzige Kategorie mit einem Gewinn (Deckungsgrad 106 %), während die anderen DRG-Gruppen einen Deckungsgrad zwischen 71 und 88 % aufwiesen (Abb. 2 und Zusatzmaterial online: Tab. E). Zugleich erwiesen sich alle anderen Fälle mit IPS-Aufenthalt als defizitär.

Die univariaten Zusammenhänge mit den erhobenen Kostenangaben sind im Zusatzmaterial online: Tab. F, partialisiert nach Versicherungsstatus, dargestellt. Dabei korrelierten neben längerem Spitalaufenthalt und den in Tab. 1 aufgezeigten Einflussfaktoren v. a. Extremitätenverletzung und die Notwendigkeit von Operationen mit höheren Verlusten. Das Vorliegen eines SHT korrelierte mit weniger Verlust, unabhängig vom Schweregrad generierten jedoch alle Mono-SHT (*n* = 102) im Durchschnitt ein Defizit (Zusatzmaterial online: Abb. B).

In der multivariaten Analyse erklärten die Parameter IPS-Aufenthalt, durchgeführte Operation, Thoraxverletzung, Nichttraumatologie- und SR-Fall zusammen 34 % (korr. R^2^) der Varianz erzielter DRG-Erlöse (Zusatzmaterial online: Tab. G). Die Tatsache eines anschließenden Rehabilitationsaufenthalts erhöhte dies um weitere 8 %.

Hingegen beschrieben die untersuchten Variablen zusammen 24 % Varianz des Deckungsgrades (Tab. 2). Ein größerer Verlust korrelierte v. a. mit dem Faktum einer SR- oder IPS-Behandlung, dagegen neurochirurgische Fallführung, eine Thoraxverletzung, und das Versterben im Krankenhaus eher zu einem Gewinn für das Spital führte: Diese Variablen erklärten zusammen 11 % der Varianz. Ein höherer Case-mix-Index sowie das Vorliegen einer Zusatzversicherung addierten weitere 13 % Varianz bzgl. eines höheren Deckungsgrades. Wurde statt der detaillierten AIS-Verletzungen allein die Tatsache einer (HSM-)Schwerverletzung ins multivariable Modell aufgenommen, so zeigte diese keine signifikante Assoziation mit dem Gesamtgewinn bzw. -verlust (Daten nicht gezeigt).

**Table 2:** 

Modell	Variable	B	β	*p*	Korrigiertes R^2^, Gesamtmodell	R^2^-Änderung pro Schritt
–	Konstante	−0,095	–	0,000	–	–
1a	Schockraumfall	−0,073	−0,185	0,000	0,05	0,05
b	Fach OE Neurochirurgie	0,053	0,132	0,002	0,06	0,01
c	Patient auf IPS	−0,133	−0,338	0,000	0,08	0,02
d	AIS3, Thorax	0,013	0,085	0,040	0,10	0,02
2	Verstorben im Krankenhaus	0,081	0,116	0,004	0,11	0,01
3a	Case Mix-Index	0,027	0,312	0,000	0,18	0,07
b	Zusatzversicherung	0,125	0,246	0,000	0,24	0,06

*B* Regressionskoeffizient B, β und entsprechender *p*-Signifikanzwert des Gesamtmodells, *R*^*2*^ Erklärungsstärken und Signifikanz pro Modell nach Hinzunahme einer Variable, *R*^*2*^*-Änderung* Verbesserung des Modells pro Schritt

## Diskussion

Diese erstmalige Kosten‑/Erlösanalyse prospektiv konsekutiv erfasster Patienten, welche in einem Schweizer Traumazentrum notfallmäßig bei V. a. Schwerverletzung bzw. aufgrund eines signifikanten Traumas behandelt worden waren, ergab drei Hauptresultate:

Erstens erwies sich die notfallmäßige Versorgung als „finanzielles Verlustgeschäft“. Letztlich deckten nur Zusatzversicherte sowie intensivmedizinische Komplexbehandlungs- bzw. PT- & SHT-DRG-Fälle die bilanzierten Spitalausgaben. Ein durchschnittlicher Verlust von 3493.- pro Patient bedeutete für das Krankenhaus ein Jahresdefizit von fast 1,8 Mio. CHF. Dies entspricht einem Deckungsgrad von 86 %, wobei 66 % der Fälle zu einem Verlust führten. Selbst wenn indirekte Kosten außen vor gelassen werden [1, 5, 7, 21, 22, 27, 30], wird die initiale Schwerverletztenbehandlung in allen internationalen Angaben, auch unter Anwendung der DRG-Entgelte, als defizitär beschrieben [26]. Erstaunlicherweise scheint die aktuelle Swiss-DRG-Entgeltung die Aufwände eines Schweizer Traumazentrums noch weniger zu decken, als dies für Deutschland der Fall ist [12, 27] und sich, im Gegensatz zu Deutschland, über die letzten Jahre auch nicht verbessert zu haben [14, 27]. Die Tatsache eines ähnlich hohen Defizits pro Schwerverletztem des Universitätsspital Zürich im Jahr 2014 [27] spricht dafür, dass dies letztlich DRG-bedingt und nicht einer speziell hohen Kostenproblematik unseres Krankenhauses zuzuschreiben ist. Angesichts eines nur 3 % höheren Tarifs (CHF 300,- pro Case-Mix-Punkt) für speziell Unfallversicherte (ca. ein Drittel aller Verletzten) gegenüber den mehrheitlich obligatorisch krankenversicherten Verletzten fand sich diesbzgl. kein relevanter Einfluss auf den ermittelten Kostendeckungsgrad. Grundsätzlich erscheint im Sinne eines „best practice“-Ansatzes [33] zukünftig eine Sollkostenkalkulation unter Einbezug validierter Qualitätsindikatoren [19] bzw. möglichst Risiko-adaptierter Outcome-Erfassung [2, 16, 18] notwendig.

Zweitens erbrachte die uni- und multivariate Detailanalyse unterschiedlich starke Korrelationen der analysierten Patienten‑, Unfall‑, Behandlungs- und Outcome-Daten mit den erhobenen Controlling-Angaben, bei zugleich sehr beschränkten Einflüssen auf die Krankenhausertragszahlen. Außer bei Zusatzversicherten gingen höhere Erlöse nicht grundsätzlich mit Gewinn oder niedrigeren Verlusten einher. So erklärten IPS-Aufenthalt oder SR-Versorgung zwar signifikant höhere DRG-Erlöse, letztlich aber eben auch signifikant höhere Gesamtverluste. Unter DRG-Aspekten besonders erstaunlich fanden wir die Tatsache, dass selbst „under-/inlier“ in 63 % der Fälle ein Defizit generierten.

Die Notwendigkeit einer detaillierten Analyse zeigt sich sehr gut am Beispiel der IPS-Behandlung schwerer Verletzter: Nur wenn diese zu einer Abrechnung des jeweiligen Falles über eine intensivmedizinische Komplexbehandlungs- oder Polytrauma & SHT-DRG führte, resultierte gesamthaft ein Gewinn. Wurde die IPS-Behandlung via einer anderen Haupt-DRG abgerechnet, ergaben sich signifikante Verluste für die Behandlung der jeweiligen Patientengruppen. Diese Beobachtung deckt sich mit neueren Angaben aus Deutschland [12, 29], mit der resultierenden Forderung nach Zuordnung aller Schwerverletzten mit ISS >15 zu einer besser entgoltenen „Polytrauma-DRG“.

Die typischen, mit hohem Aufwand verbundenen, Traumazentrumaufgaben Primärversorgung, SR-, Schwerverletzten- oder IPS-Behandlung waren mehrheitlich defizitär. Neuere Publikationen bestätigen dies z. B. für die Untergruppe im SR betreuter Patienten mit leichtem SHT eines dt. Traumazentrums [34], dies im Gegensatz zu anderen Bereichen, wo leitliniengerecht erbrachte Aufwände in der Zentrumsbehandlung des akuten Schlaganfalls oder akutgeriatrischer Betreuung gemäß DRG adäquat bezahlt werden und Gewinne erzielt werden können. Der in der initialen Notfallversorgung ähnlich hohe klinische Aufwand für den potenziell wie für den effektiv Schwerverletzten [20], im Sinne einer „im Zweifel ‚over‘- statt ‚undertriage‘“ [3] wird somit finanziell bestraft. Auch der Umstand, dass im Spital verstorbene Verletzte weniger Defizit generieren, erscheint paradox, wurde allerdings auch für ein dt. Traumazentrum nachgewiesen [17].

Aufgrund der komplexen Zusammenhänge zahlreicher potenzieller Einflussfaktoren führten wir in dieser Thematik selten publizierte multivariate Analysen [5, 7] durch. Während 43 % der Varianz der erhobener DRG-Erlöse erklärt werden konnten (mehr als bisher beschrieben [5]), ließ sich der Kostendeckungsgrad nur zu 24 % mit den untersuchten Variablen erklären. Wird zudem der 13 % umfassende Anteil aufgrund des höheren Case-Mix-Index und zusatzversicherter Patienten abgezogen, bleiben gerade noch 11 %: Spezifisch neurochirurgische Behandlungsfälle sowie schwere Thoraxverletzungen erklärten dabei zu je 1–2 % eher Gewinn. Hingegen waren IPS-Aufenthalt (2 %) sowie v. a. der SR-Einsatz (5 %) mit mehr Verlust verbunden. Diese niedrigen Varianzen weisen auf unzureichend erklärbare ursächliche Faktoren hin. Es zeigt sich allerdings, dass v. a. weniger schwer Verletzte bzw. bei niedrigem Case-Mix-Index zu schlecht abgegolten werden, d. h. typischerweise eine SR-Behandlung, welche, „ex post“ gesehen, aufgrund der Verletzungsschwere bzw. des Gesamtzustands des Patienten nicht unbedingt nötig gewesen wäre. Die beobachtete grundlegende bzw. systembedingte Unterdeckung ist sehr unbefriedigend und lässt die bisherige DRG-Konzeption anzweifeln. Bereits in den frühen 1990er-Jahren publizierten US-amerikanische Traumazentren klinisch relevante Entgeltverbesserungsvorschläge unter Einbezug von Komorbidität und Outcome der Patienten, angesichts der abschreckenden Unterbezahlung für derartige Notfallzentrumsleistung [32]. Strausberg betont, dass im DRG-System die verursachungsgerechte Vergütung sichergestellt werden soll, im Sinne einer Kostenerstattung für diejenigen Krankenhäuser, welche sich im unauffälligen Mittelfeld der Kosten befinden [33].

Drittens ergab sich bei nur 42 % der Zusatzversicherten gegenüber 71 % der Allg.-Versicherten ein Verlust, wobei bei Ersteren sogar ein Gewinn von CHF 1318 pro Patient resultierte (Deckungsgrad 104 %). Somit senkten die 18 % Zusatzversicherten das bei alleinigen DRG-Erträgen ansonsten generierte Spitaldefizit von über 2,3 Mio. CHF um sicher 500.000.-. Multivariat erklärte die Tatsache einer Zusatzversicherung zusätzliche 6 % zu den nur 18 %, mit welchen sich ansonsten die Höhe jeweiliger Spitalverluste bzw. allenfalls Gewinne durch die untersuchten Variablen, inklusive des Case-Mix-Index, beschreiben ließ. Vergleichbare Literaturdaten zur Zusatzhonorierung initialer SR- bzw. Schwerverletztenversorgung über die Grundversicherung hinaus lassen sich nicht finden; neue Arbeiten beschränken sich entweder auf reine DRG-Erlöse [7, 13, 27, 29, 34] oder machen keine Angaben hierzu [11]. Hinzu kommt die international schwierige Vergleichbarkeit der Entgeltsysteme. So benutzt das ursprünglich auf dem deutschen DRG-System basierende Schweizer System andere Prozedurenkataloge (D: OPS vs. CH: CHOP), und die Kostengewichte wurden aufgrund Schweizer Daten berechnet und sind daher nicht mit den deutschen Kostengewichten (CW) identisch [10, 24]. Zudem müssen gemäß gesetzlicher Regelung im Schweizer DRG-System – im Gegensatz zu Deutschland – zukünftige Investitionen durch Einnahmen aus der Patientenversorgung gedeckt sein, und es dürfen offiziell keine Quersubventionen oder Defizitgarantien seitens z. B. der Kantone erfolgen [10]. Umso mehr stellt sich die grundsätzliche Frage, inwieweit die unsererseits erstmals für ein Traumazentrum in der Schweiz nachgewiesene Querfinanzierung seitens Zusatzversicherter im Bereich notfallmäßiger Zentrumsversorgung meist öffentlicher Träger angebracht bzw. erlaubt ist.

### Limitationen

Die Untersuchung war monozentrisch und auf ein Jahr begrenzt, mit entsprechend beschränkter Fallzahl, v. a. auch für Detailanalysen seltener Untergruppen. Die beschriebenen Angaben beruhen auf Controlling-Daten, welche z. B. keine Echterfassung personeller Arbeitsaufwände im SR etc. umfassen, womit die effektiven Verluste eher noch höher einzuschätzen sind [17]. Bzgl. DRG-Erlösen gilt es, die länderspezifischen Versionen zu berücksichtigen, allerdings erlauben die Angaben gemäß schweizweit angewandtem REKOLE©-Verfahren zumindest zwischen Schweizer Spitälern vergleichbare Untersuchungen, welche allerdings bisher nicht durchgeführt wurden [27, 34].

## Fazit für die Praxis

Angesichts der jährlichen Falldefizite in Millionenhöhe eines Schweizer Traumazentrums für die Notfallversorgung potenziell schwer bzw. im SR versorgter Verletzter sollten sich die zuständigen Verantwortlichen bzw. Fachgesellschaften rasch für eine Verbesserung der betreffenden (DRG-)Entgelte einsetzen. Obwohl erst vor wenigen Jahren aus dem deutschen DRG-System entwickelt, weist diese Arbeit angesichts mehrheitlich derart negativer Kostendeckungsgrade auf noch schlechtere aktuelle Schweizer DRG-Ansätze als vergleichbare deutsche hin. Zudem ist der Querfinanzierungsgrad seitens Zusatzversicherter zu hinterfragen. Beispielsweise bzgl. ‚Stroke‘-Management oder im Rahmen akutgeriatrischer Betreuung („Komplexbehandlung“) scheinen die seitens der Fachrichtlinien eingeforderten hohen Struktur‑, Prozess- und Outcome-Aufwände adäquater berücksichtigt bzw. finanziell entgolten zu werden. Zudem erscheint aus klinischer Sicht eine Sollkostenkalkulation unter Einbezug validierten Qualitätsindikatoren bzw. möglichst risikoadaptierter Outcome-Erfassung im Sinne eines „Best-practice“-Ansatzes überfällig.

## Supplementary Information





## References

[1] Anders B, Ommen O, Pfaff H (2013). Direct, indirect, and intangible costs after severe trauma up to occupational reintegration—An empirical analysis of 113 seriously injured patients. Psychosoc Med.

[2] Attenberger C, Amsler F, Gross T (2012). Clinical evaluation of the Trauma Outcome Profile (TOP) in the longer-term follow-up of polytrauma patients. Injury.

[3] Braken P, Amsler F, Gross T (2018). Simple modification of trauma mechanism alarm criteria published for the TraumaNetwork DGU® may significantly improve overtriage—A cross sectional study. Scand J Trauma Resusc Emerg Med.

[4] Busse R, Geissler A, Aaviksoo A (2013). Diagnosis related groups in Europe: moving towards transparency, efficiency, and quality in hospitals?. BMJ.

[5] Christensen MC, Ridley S, Lecky FE (2008). Outcomes and costs of blunt trauma in England and Wales. Crit Care.

[6] Cotte J, Courjon F, Beaume S (2016). Vittel criteria for severe trauma triage: Characteristics of over-triage. Anaesth Crit Care Pain Med.

[7] Curtis K, Lam M, Mitchell R (2014). Acute costs and predictors of higher treatment costs of trauma in New South Wales, Australia. Injury.

[8] Curtis K, Lam M, Mitchell R (2014). Major trauma: the unseen financial burden to trauma centres, a descriptive multicentre analysis. Aust Health Rev.

[9] Eastham JN, Steinwachs DM, Mackenzie EJ (1991). Trauma care reimbursement: Comparison of DRGs to an injury severity-based payment system. J Trauma.

[10] Fässler M, Wild V, Clarinval C (2015). Impact of the DRG-based reimbursement system on patient care and professional practise: perspectives of Swiss hospital physicians. Swiss Med Wkly.

[11] Fountain DM, Kolias AG, Laing RJ (2017). The financial outcome of traumatic brain injury: a single centre study. Br. J Neurosurg.

[12] Franz D, Lefering R, Siebert H (2013). Die Herausforderung der sachgerechten Vergütung von Scherverletzten im deutschen DRG-System. Gesundheitswesen.

[13] Garving C, Santosa D, Bley C et al (2014) Cost analysis of emergency room patients in the German diagnosis-related groups system. Unfallchirurg 117:716–722 10.1007/s00113-013-2405-2 23928797

[14] Gross T, Amsler F (2016). Long-term outcome following multiple trauma in working age. Unfallchirurg.

[15] Gross T, Braken P, Amsler F (2020). Trauma center need: the American College of Surgeons’ definition in contrast to Swiss highly specialized medicine regulations—A Swiss trauma center perspective. Eur J Trauma Emerg Surg.

[16] Gross T, Morell S, Scholz SM (2019). The capacity of baseline patient, injury, treatment and outcome data to predict reduced capacity to work and accident insurer costs—A Swiss prospective 4-year longitudinal trauma centre evaluation. Swiss Med Wkly.

[17] Grotz M, Schwermann T, Lefering R (2004). DRG reimbursement for multiple trauma patients—a comparison with the comprehensive hospital costs using the German trauma registry. Unfallchirurg.

[18] Hashmi ZG, Schneider EB, Castillo R (2014). Benchmarking trauma centers on mortality alone does not reflect quality of care: implications for pay-for-performance. J Trauma Acute Care Surg.

[19] Hörster AC, Kulla M, Bieler D (2020). Empirische Überprüfung der Qualitätsindikatoren für Schwerverletzte im TraumaRegister DGU. Unfallchirurg.

[20] Lang J, Dallow N, Lang A (2014). Inclusion of ‘minor’ trauma cases provides a better estimate of the total burden of injury: Queensland Trauma Registry provides a unique perspective. Injury.

[21] Lee H, Croft R, Monos O (2018). Counting the costs of major trauma in a provincial trauma centre. N Z Med J.

[22] Lefering R, Mahlke L, Franz D (2017). Der Kostenschätzer im TraumaRegister DGU. Unfallchirurg.

[23] Mackenzie EJ, Steinwachs DM, Ramzy AI (1991). Trauma case mix and hospital payment: the potential for refining DRGs. Health Serv Res.

[24] Mahlke L, Lefering R, Siebert H et al (2013) Abbildung von Schwerverletzten im DRG-System. Chirurg 84:978–986 10.1007/s00104-013-2490-3 23512224

[25] Marzi I, Lustenberger T, Störmann P (2019). Steigender Vorhalteaufwand für den Schockraum. Unfallchirurg.

[26] Mehra T, Koljonen V, Seifert B (2015). Total inpatient treatment costs in patients with severe burns: towards a more accurate reimbursement model. Swiss Med Wkly.

[27] Moos RM, Sprengel K, Jensen KO (2016). Reimbursement of care for severe trauma under SwissDRG. Swiss Med Wkly.

[28] Obertacke U, Neudeck F, Wihs HJ (1997). Kostenanalyse der Primärversorgung und intensivmedizinischen Behandlung polytraumatisierter Patienten. Unfallchirurg.

[29] Qvick B, Buehren V, Woltmann A (2012). Is polytrauma affordable these days? G-DRG system vs per diem charge based on 1,030 patients with multiple injuries. Unfallchirurg.

[30] Rowell D, Connelly L, Webber J (2011). What are the true costs of major trauma?. J Trauma.

[31] Schwab CW, Young G, Civil I (1988). DRG reimbursement for trauma: the demise of the trauma center (the use of ISS grouping as an early predictor of total hospital cost). J Trauma.

[32] Siegel JH, Shafi S, Goodarzi S (1994). A quantitative method for cost reimbursement and length of stay quality assurance in multiple trauma patients. J Trauma.

[33] Stausberg J (2012). Ist ein Polytrauma heutzutage noch bezahlbar?. Unfallchirurg.

[34] Verboket R, Verboket C, Schöffski O (2019). Kosten und Erlöse von über den Schockraum eingelieferten Patienten mit leichtem Schädel-Hirn-Trauma. Unfallchirurg.

