# Supplementary material for: Emergency room and major trauma treatment is a “loss-making business”: A Swiss trauma center experience with current DRG reimbursement
Source: Unfallchirurg. 2020 Dec 18;124(9):747–54. [Article in German] doi: 10.1007/s00113-020-00937-w (PMC8397679; doi:10.1007/s00113-020-00937-w)
Supplement: Supplementary file 1 [file 113_2020_937_MOESM1_ESM.pdf]

Suppl. Tabelle A Detaillierte Patienten-, Unfall-, Behandlungs- und Outcome- Charakteristika des Studienkollektives

	N	MW	SD	Min	Max	25%	Median	75%
Alter bei Unfall	513	57.26	25.05	0	98	39.5	62	78
1. syst. Blutdruck	511	140.30	28.84	0	210	120	140	159
1. GCS	512	13.23	3.31	3	15	14	15	15
ISS	513	13.33	9.27	0	75	8.5	10	17
NISS	513	17.62	12.41	0	75	9	16	25
GAP	510	20.14	3.75	6	24	19	21	23
Letalitätsrate (Spital)	513	8.58	28.03	0	100	0	0	0
RISC2 (%)	513	8.94	16.05	0.04	99.86	0.41	1.98	9.11
Age unadjusted Charlson Score	513	0.89	1.48	0	9	0	0	1
Age adjusted Charlson Score	513	3.16	2.67	0	13	1	3	5
AIS1 Kopf/Hals	513	2.08	1.69	0	6	0	2	3
AIS2 Gesicht	513	0.29	0.70	0	3	0	0	0
AIS3 Thorax	513	0.69	1.27	0	5	0	0	1
AIS4 Abdomen	513	0.43	0.99	0	5	0	0	0
AIS5 Extremitäten	513	0.79	1.18	0	5	0	0	2
AIS6 Weichteile	513	0.47	0.54	0	3	0	0	1
AIS Schädel/Hirn	513	2.02	1.70	0	5	0	2	3
AIS Extremitäten	513	0.70	1.09	0	4	0	0	2
Case Mix Index	513	1.93	2.25	0.23	18.88	0.80	1.06	2.32
Pflegeaufwand (LEP©) Total	513	3961	4690	155	39035	1094	2196	4854
Hospitalisationstage	513	8.0	7.1	1	42	3	6	12
GOS - Abschluss	493	4.15	1.17	1	5	4	4	5

	N tot	N ja	%
Geschlecht weiblich	513	190	37.0%
Kind (<16 Jahre)	513	39	7.6%
Unfallmechanismus Energie hoch	508	193	38.0%
Versorgung sekundär	513	156	30.4%

BD syst ≤ 90	511	22	4.3%
1.GCS < 13	512	93	18.2%
Chirurg. Not-Eingriff inkl. Präklinik (Thoraxdrainage, Not-OP) oder Damage Control-Eingriff	513	81	15.8%
Chirurg. Eingriff inkl. Präklinik (Txoraxdrainage, Not-OP oder OP)	513	215	41.9%
Intubiert (Präklinik bis IPS, ohne OP)	513	92	17.9%
NISS>=8 & Hospitalisation <24h nach Unfall	513	373	72.7%
Schockraum-Fall	513	281	54.8%
ISS ≥16	513	182	35.5%
Mehrfachverletzung	513	333	64.9%
Polytrauma (ISS >16 & 2 AIS-Regionen >0)	513	132	25.7%
HSM (ISS ≥20 (Kinder ≥16) oder AIS Schädel/Hirn≥3)	513	250	48.7%
AIS1 Kopf/Hals >2	513	230	44.8%
AIS2 Gesicht >2	513	13	2.5%
AIS3 Thorax >2	513	87	17.0%
AIS4 Abdomen >2	513	35	6.8%
AIS5 Extremitäten >2	513	61	11.9%
AIS6 Weichteile >2	513	1	0.2%
AIS Schädel/Hirn >2	513	226	44.1%
AIS Extremitäten >2	513	50	9.7%
Unfallmechanismus penetrierend	512	14	2.7%
Maximaler AIS >3	513	140	27.3%
GOS<5 (mind. nicht gut erholt)	493	248	50.3%
Rehabilitationsaufenthalt nach Austritt	513	129	25.1%
Verstorben im Krankenhaus	513	44	8.6%

SD: Standard Deviation; GCS: Glasgow Coma Scale; (N)ISS: (New) Injury Severity Score; GAP: GCS + Age and Arterial Pressure; RISC2 = Revised Injury Severity Classification (Version 2); AIS: Abbreviated Injury Scale; LEP: LeistungsErfassung von Pflegeleistungen gesamthaft, http://www.lep.ch); GOS: Glasgow Outcome Scale; BD: Blutdruck systolisch; OP: Operation; HSA: Hochspezialisierte Medizin (CH), Kriterien "Schwerverletzt" erfüllt

Suppl. Tabelle B: Kosten-Matrix nach REKOLE©

DB I	DB II	DB III Rekole
**Einzelkosten**	**Gemeinkosten**	**Infrastruktur- bzw. Anlagekosten Rekole**
Medizinischer Bedarf	10 Patientenadministration	05 AN Miete
101 Arzneimittel	20 OP Saal	02 AN Rekole bis 0
102 Blut und Blutprodukte	23 Anästhesie	03 AN Rekole kleiner 0
103 Medizinisches Material	24 IPS und IMCU	07 AN Fremdfinanzierung Rekole
104 Implantate	25 Notfall	
105 Med., therap., diag. Fremdleistungen	26 Bildgebene Verfahren	
Arzthonorarkosten	27 Gebärsaal	
Übrige Einzelkosten	28 NUK und RAO	
	29 Labor	
	30 Dialyse	
	31 Ärzteschaften	
	212 Ärzteschaften des OP-Saals	
	232 Ärzteschaften der IPS	
	252 Ärtzteschaften des Notfalls	
	310 Ärzteschaften, Aktivitäten 1-5	
	362 Ärzteschaften der medizinischen und therapeutischen Diagnostik	
	32 Physiotherapie	
	33 Ergotherapie	
	34 Logopädie	
	35 Nichtärztl. Therapien und Beratungen	
	38 Anerk. IPS u. IMCU	
	39 Pflege	
	41 Hotellerie-Zimmer	
	42 Hotellerie-Küche	

43 Hotellerie-Service
44 Übr. Leistungserbringer
45 Pathologie
47 Forschung und univers. Lehre
77 Ambulanz und Rettungsdienst

Suppl. Tabelle C Vergleichende Studienkollektiv- bzw. Spital-Kosten-/ Erlöse-Angaben bzgl. Allgemein- vs. Zusatzversicherte

Zusatzversicherung	Total (N=513)	Allgemein (N=421)	Privat / Halbprivat (N=92)		
	Mean (SD)	Mean (SD)	Mean (SD)	R^2	P*
Alter bei Unfall	57.3 (25)	56.1 (24.8)	62.7 (25.6)	0.01	.021
ISS	13.3 (9.3)	12.8 (8.9)	15.9 (10.6)	0.02	.004
RISC2 in %	8.9 (16.1)	8.5 (15.5)	10.9 (18.5)	0.00	.191
Verstorben Rate in %	8.6	8.1	10.9	0.00	.387
Overlier (gemäss DRG) in %	15.8	16.4	13.0	0.00	.426
Gesamt-Erlöse	21474 (24676)	18663 (21941)	34339 (31627)	0.06	.000
DRG-Erlöse	18953 (22109)	18193 (21775)	22434 (23386)	0.01	.096
Einzelkosten	-2551 (4181)	-1851 (2747)	-5752 (7135)	0.13	.000
DB I	18924 (22186)	16812 (20511)	28587 (26694)	0.04	.000
Individueller Deckungsgrad DB I *	27.63 (45.57)	31.61 (49.32)	9.44 (7.07)	0.03	.000
DB I Patienten mit Gewinn	0.99	0.99	1	0.00	.294
Gemeinkosten	-19848 (20765)	-18871 (20255)	-24323 (22536)	0.01	.022
Einzel- + Gemeinkosten	-22399 (23534)	-20722 (22026)	-30075 (28363)	0.02	.001
DB II	-925 (10539)	-2059 (9645)	4265 (12744)	0.05	.000
Individueller Deckungsgrad DB II *	1.03 (0.48)	0.99 (0.49)	1.23 (0.36)	0.06	.000
DB II Patienten mit Gewinn	0.43	0.38	0.70	0.06	.000
Anlagenutzungskosten	-2569 (2751)	-2486 (2812)	-2947 (2433)	0.00	.146
Gesamtkosten	-24968 (26147)	-23208 (24737)	-33021 (30699)	0.02	.001
DB III	-3493 (11211)	-4545 (10499)	1318 (13035)	0.04	.000
Deckungsgrad *	86.0%	80.4%	104.0%	0.07	.000
DB III Patienten mit Gewinn	0.34	0.29	0.58	0.05	.000

** Signifikanz gemäss logarithmierter Werte. Die Stärke der jeweiligen Beziehung und Signifikanz (R^2 bzw. P) wird durch die Intensität der jeweiligen Farbmarkierung illustriert.*

SR: Standard Deviation; ISS: Injury Severity Score; RISC2: Revised Injury Severity Classification Score 2; DRG: diagnosis related groups; DB: Deckungsbeiträge gemäss REKOLE©- Abrechnung; DB I = Erlöse minus Einzelkosten; DB II = DB I minus Betriebskosten (= Einzel- plus Gemeinkosten; DB III: Gesamt-Gewinn/ Verlust (=DB II minus Anlagenutzungskosten)

Suppl. Tabelle D Kostendaten je nach Kombination Schwerverletzung (HSM) und / oder SR-versorgung (ja/ nein)

SR / Schwerverl 4k	Total (N=513)	G1: kein SR / keine Schwerverl (N=78)	G2: SR / keine Schwerverl (N=185)	G3: kein SR / Schwerverl (N=154)	G4: SR / Schwerverl (N=96)	Einzelvergleiche p mit Bonferroni-Korrektur						
	Mean (SD)	Mean (SD)	Mean (SD)	Mean (SD)	Mean (SD)	p	G1 vs. G2	G1 vs. G3	G1 vs. G4	G2 vs. G3	G2 vs. G4	G3 vs. G4
Alter bei Unfall	57.3 (25)	66.6 (20.6)	43.3 (24)	68.4 (21.3)	58.9 (23.1)	.000	.000	1.000	.152	.000	.000	.008
ISS	13.3 (9.3)	10.1 (3.7)	6.8 (5.2)	16.5 (7)	23.3 (10.6)	.000	.002	.000	.000	.000	.000	.000
RISC2 (%)	8.9 (16.1)	5.9 (9.8)	2.4 (7.9)	11.5 (12.7)	19.9 (26.6)	.000	.469	.041	.000	.000	.000	.000
Verstorben Rate	8.6 (28)	11.5 (32.2)	3.8 (19.1)	5.8 (23.5)	19.8 (40.1)	.000	.222	.819	.296	1.000	.000	.001
Overlier (gemäss DRG)	0.16 (0.37)	0.08 (0.268)	0.18 (0.38)	0.14 (0.35)	0.208 (0.408)	.089	.236	1.000	.109	1.000	1.000	1.000
Erlöse	21474 (24676)	22118 (15803)	17172 (22542)	17742 (18570)	35230 (35911)	.000	.745	1.000	.002	1.000	.000	.000
DRG-Erlöse	18953 (22109)	19284 (13600)	15456 (21274)	15169 (13138)	31495 (33524)	.000	1.000	.990	.001	1.000	.000	.000
Gesamtkosten	-24968 (26147)	-25565 (17909)	-21812 (26376)	-20253 (22150)	-38127 (32501)	.000	1.000	.797	.007	1.000	.000	.000
Gewinn/Verlust (DB III)	-3493 (11211)	-3447 (8259)	-4640 (10598)	-2511 (8719)	-2898 (16662)	.336	1.000	1.000	1.000	.493	1.000	1.000
Deckungsgrad *	86.0%	86.5%	78.7%	87.6%	92.4%	.000	.011	1.000	.320	.000	1.000	.013
Patienten mit Gewinn	0.34 (0.48)	0.37 (0.49)	0.26 (0.44)	0.45 (0.5)	0.29 (0.46)	.003	.553	1.000	1.000	.002	1.000	.064

** Signifikanz gemäss logarithmierter Werte. Die Bonferroni-korrigierte Signifikanz (p) wird durch die Farbmarkierung illustriert.*

SR: Schockraumbetreuung; Schwerverl: Schwerverletzte gemäss HSM- (Hochspezialisierte Medizin) Definition der Schweiz
SD: Standard Deviation; ISS: Injury Severity Score; RISC2: Revised Injury Severity Classification Score 2; DRG: diagnosis related groups;
DB: Deckungsbeiträge gemäss REKOLE©- Abrechnung; DB III: Gesamt-Gewinn/ Verlust (=DB II minus Anlagenutzungskosten)

Supp. Tab. E Vergleichende Kostendaten je nach gruppierten Haupt-DRGs

Mean (SD)	Total (N=513)	PT (N=70)	IPS od (PT & SHT) (N=36)	SHT (N=187)	WS (N=73)	Extr (N=48)	andere (N=99)	p
Alter bei Unfall	57.3 (25)	58 (23.6)	65.3 (16.6)	57.2 (27.7)	60 (21)	53.2 (24.3)	53.9 (25.9)	.175
ISS	13.3 (9.3)	21.8 (11.8)	18.3 (11.8)	10.4 (6.3)	18.3 (8.2)	9.4 (5)	9.2 (6.7)	.000
RISC2 (%)	8.9 (16.1)	15.1 (24.7)	15.1 (19.3)	7.6 (14.1)	12 (16.3)	3.3 (6.2)	5.3 (10.7)	.000
Verstorben Rate	8.6 (28)	24.3 (43.2)	19.4 (40.1)	4.8 (21.5)	6.9 (25.4)	0 (0)	6.1 (24)	.000
Overlier gemäss DRG	0.16 (0.37)	0.24 (0.43)	0.11 (0.32)	0.19 (0.39)	0.16 (0.37)	0.08 (0.28)	0.09 (0.29)	.052
Erlöse	21474 (24676)	22439 (13969)	86025 (39308)	8340 (5785)	29198 (17144)	18341 (10239)	17952 (15495)	.000
DRG-Erlöse	18953 (22109)	20739 (13452)	77486 (34878)	7141 (4290)	25710 (15282)	16051 (7782)	15143 (13286)	.000
Gesamtkosten	-24968 (26147)	-25886 (17990)	-80892 (45507)	-10844 (7931)	-33031 (20597)	-25885 (17780)	-24271 (21028)	.000
Gewinn/ Verlust (DBIII)	-3493 (11211)	-3448 (10239)	5133 (24249)	-2503 (5507)	-3833 (11904)	-7544 (11536)	-6319 (10097)	.000
Deckungsgrad *	86.0%	86.7%	106.3%	76.9%	88.4%	70.9%	74.0%	.000
Patienten mit Gewinn	0.34 (0.48)	0.43 (0.5)	0.56 (0.5)	0.30 (0.46)	0.44 (0.5)	0.29 (0.46)	0.23 (0.42)	.001

** Signifikanz bezieht sich auf logarithmierte Werte*

SD: Standard Deviation; ISS: Injury Severity Score; RISC2: Revised Injury Severity Classification Score 2; DRG: diagnosis related groups;
DB: Deckungsbeiträge gemäss REKOLE©- Abrechnung; DB III: Gesamt-Gewinn/ Verlust

Suppl. Tab F Einfluss auf Kosten (nur relevante Variabeln, partialisiert nach Zusatzversicherung

Variablen	Erlöse	Einzelkosten	Gesamtkosten	DB III	Deckungsgrad log	Patienten mit Gewinn
**Alter bei Unfall**	0.11**	-0.05	-0.12**	-0.04	0.07	0.04
**1. syst. Blutdruck**	-0.01	0.04	0.03	0.04	0.05	0.06
**1. GCS**	-0.21***	-0.01	0.17***	-0.06	0.03	-0.06
**ISS**	0.28***	-0.17***	-0.26***	-0.02	0.03	0.05
**NISS**	0.28***	-0.13**	-0.25***	0.02	0.01	0.02
**GAP**	-0.22***	-0.01	0.18***	-0.05	0.01	-0.05
**Verstorben Rate**	0.01	-0.02	0.01	0.05	0.06	0.07
**RISC2 (%)**	0.1*	-0.06	-0.1*	-0.01	0.01	0.02
**Age unadjusted Charlson Score**	0.06	-0.06	-0.07	-0.02	0.06	0.04
**Age adjusted Charlson Score**	0.1*	-0.05	-0.1*	-0.02	0.08	0.05
**AIS1 Kopf/Hals**	0	0.08	0.03	0.07	0.06	0.08
**AIS2 Gesicht**	0.14**	-0.04	-0.14***	-0.04	-0.07	-0.05
**AIS3 Thorax**	0.24***	-0.08	-0.19***	0.06	0.04	0.06
**AIS4 Abdomen**	0.08	-0.17***	-0.1*	-0.05	-0.02	-0.04
**AIS5 Extremitäten**	0.19***	-0.25***	-0.26***	-0.20***	-0.07	-0.09*
**AIS6 Weichteile**	-0.05	0.04	0.02	-0.04	-0.11*	-0.09*
**AIS Extremitäten**	0.2***	-0.26***	-0.28***	-0.21***	-0.09*	-0.13**
**LEP Total**	0.84***	-0.47***	-0.92***	-0.34***	-0.11*	-0.13**
**Hospitalisationstage**	0.7***	-0.55***	-0.81***	-0.40***	-0.14**	-0.19***
**GOS - Abschluss**	-0.24***	0.16***	0.23***	0.03	0	0
**Geschlecht weiblich**	-0.04	0.03	0.04	0	-0.03	-0.03
**Kind (<16 Jahre)**	-0.17***	0.14**	0.17***	0.03	-0.1*	-0.09*

**Unfallmechanismus Energie hoch**	0.05	-0.06	-0.05	0	-0.01	-0.03
**Versorgung sekundär**	0	0.05	0.03	0.07	0.18***	0.16***
**BD syst ≤ 90**	0.04	-0.06	-0.09*	-0.12**	-0.08	-0.11*
**1.GCS < 13**	0.19***	0.01	-0.15***	0.05	-0.06	0.05
**Chirurg. Not-Eingriff inkl. Präklinik (Thoraxdrainage, Not-OP) oder Damage Control-Eingriff**	0.42***	-0.41***	-0.4***	-0.04	-0.02	0.03
**Chirurg. Eingriff inkl. Präklinik (Thoraxdrainage, Not-OP oder OP)**	0.43***	-0.5***	-0.47***	-0.17***	-0.07	-0.07
**Intubiert (Präklinik bis IPS, ohne OP)**	0.48***	-0.26***	-0.45***	0	0	0.03
**NISS>=8 & Hospitalisation <24h nach Unfall)**	0.12**	-0.1*	-0.14***	-0.07	0.01	-0.03
**Schockraum-Fall**	0.08	-0.03	-0.1*	-0.06	-0.23***	-0.16***
**ISS ≥16**	0.26***	-0.15***	-0.25***	-0.04	-0.04	0.01
**Mehrfachverletzung**	0.03	0	-0.04	-0.01	-0.06	-0.06
**Polytrauma (ISS >16 & 2 AIS-Regionen >0)**	0.25***	-0.11*	-0.25***	-0.04	-0.05	0
**HSM**	0.11*	0.03	-0.08	0.07	0.12**	0.09*
**AIS1 Kopf/Hals >2**	0.01	0.09*	0.02	0.07	0.11*	0.11*
**AIS2 Gesicht >2**	0.04	-0.05	-0.06	-0.06	-0.09*	-0.04
**AIS3 Thorax >2**	0.17***	-0.04	-0.14**	0.03	0.02	0.06
**AIS4 Abdomen >2**	0.06	-0.16***	-0.07	-0.03	0.02	0.01
**AIS5 Extremitäten >2**	0.19***	-0.27***	-0.25***	-0.19***	-0.02	-0.08
**AIS6 Weichteile >2**	-0.03	-0.04	0.03	0	-0.07	-0.03
**AIS Schädel/Hirn >2**	0.01	0.1*	0.02	0.07	0.11**	0.11*
**AIS Untere Extremitäten >2**	0.17***	-0.31***	-0.24***	-0.19***	-0.03	-0.09*
**AIS Extremitäten >2**	0.22***	-0.32***	-0.29***	-0.21***	-0.03	-0.09*
**Unfallmechanismus penetrierend**	0.11*	-0.04	-0.15***	-0.12**	-0.08	-0.09
**Maximaler AIS >3**	0.22***	-0.11*	-0.21***	-0.02	-0.03	0
**GOS<5 (mind. nicht gut erholt)**	0.36***	-0.25***	-0.38***	-0.11*	-0.07	-0.09
**Rehabilitationsaufenthalt nach Austritt**	0.51***	-0.34***	-0.55***	-0.18***	-0.09*	-0.11**
**Verstorben im Krankenhaus**	0.01	-0.02	0.01	0.05	0.06	0.07

GCS: Glasgow Coma Scale; (N)ISS: (New) Injury Severity Score; GAP: GCS + Age and Arterial Pressure; RISC2 = Revised Injury Severity Classification (Version 2); AIS: Abbreviated Injury Scale; LEP: Pflegerischer Aufwand pro Patient und Tag (gemäß „Leistungserfassung und Prozessdokumentation im Gesundheitswesen“, http://www.lep.ch); GOS: Glasgow Outcome Scale; BD: Blutdruck systolisch; OP: Operation; HSA: Hochspezialisierte Medizin (CH), Kriterien "Schwerverletzt" erfüllt

Suppl. Tabelle G Lineare Regression der Einflussfaktoren (Patienten-, Unfall-, Behandlungs- und Outcome-Daten) auf erzielte DRG-Erlöse

Modell	Variable	B	Beta	p	Korrigiertes R^2 Gesamtmodell	R^2-Änderung pro Schritt
	Konstante	32		.990		
1	Patient auf Intensivstation	11658	0.261	.000	0.213	0.215
2	OP durchgeführt	9852	0.216	.000	0.298	0.086
3	Abbreviated Injury Score Thoraxregion	2364	0.135	.000	0.313	0.016
4	Hauptbetreuende Klinik Traumatologie	-5711	-0.128	.001	0.324	0.013
5	Schockraum-Fall	4946	0.111	.004	0.331	0.008
6	Alter bei Unfall	56	0.063	.091	0.338	0.008
7	Abbreviated Injury Score Extremitäten	1416	0.070	.084	0.344	0.007
8	Reha nach Austritt	16147	0.317	.000	0.426	0.082

B: Regressionskoeffizient B; Beta und entsprechender p-Signifikanzwert des Gesamtmodells; R^2: Erklärungsstärken und Signifikanz pro Modell nach Hinzunahme einer Variable, R2 Änderung: Verbesserung des Modells pro Schritt

Suppl. Abb. A Verteilung des Studienkollektives je nach (HSM) Schwerverletzung, SR- oder Primärversorgung (ja/ nein)

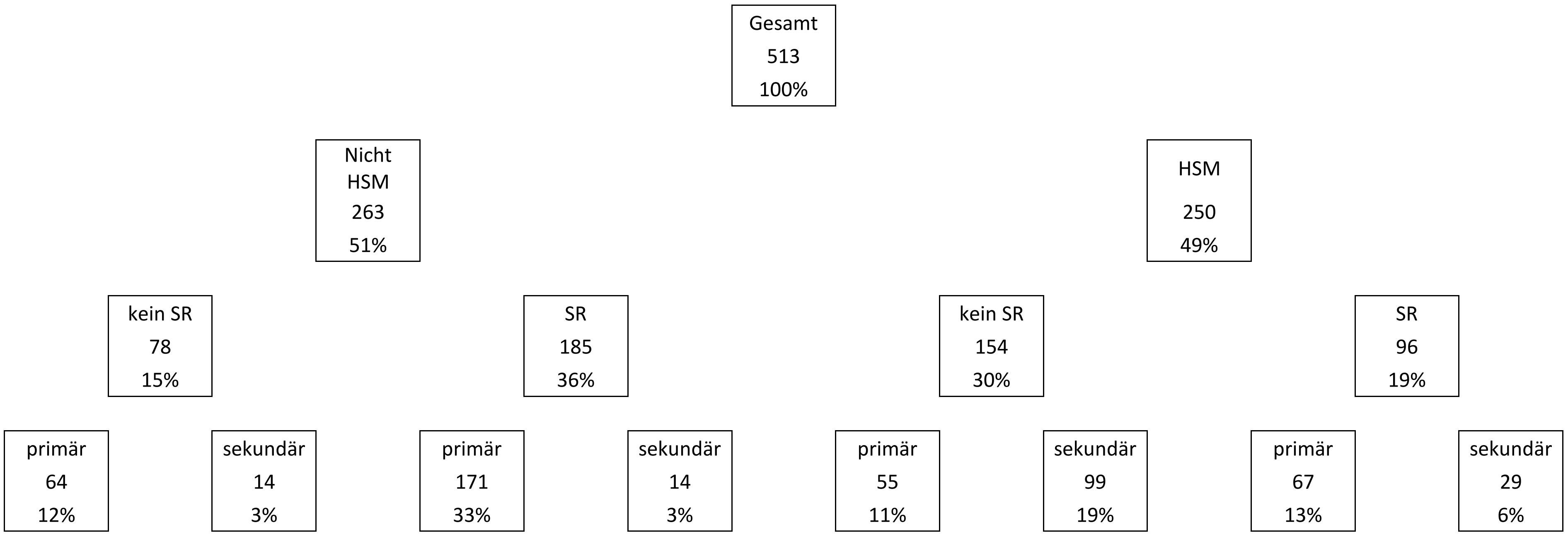

SR: Schockraumbetreuung; HSM: Schwerverletzte gemäss HSM- (Hochspezialisierte Medizin) Definition der Schweiz; primär / sekundär: primär versorgte vs. sekundär zuverlegte Patienten

Suppl. Abb. B Erlöse bzw. Gewinn-/ Verlust-Darstellung je nach Schweregrad (AIS) monotraumatisierter Kopf-/ Halsverletzter

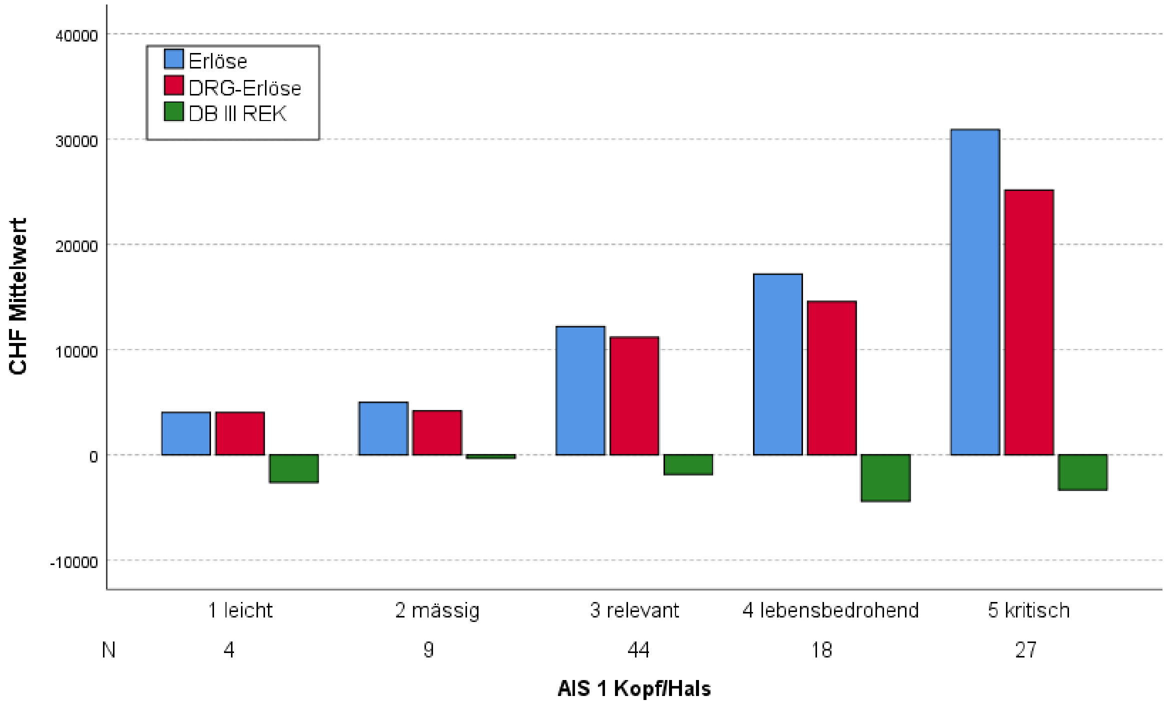

AIS: Abbreviated Injury Scale; DB III REK: Deckungsbeiträge gemäss REKOLE©-Abrechnung Gesamt-Gewinn/ Verlust
